# An evaluation of equity and equality in physical activity policies in four European countries

**DOI:** 10.1186/s12939-016-0481-y

**Published:** 2016-11-24

**Authors:** Riitta-Maija Hämäläinen, Petru Sandu, Ahmed M. Syed, Mette W. Jakobsen

**Affiliations:** 1 National Institute for Health and Welfare, Mannerheimintie 166, 00271 Helsinki, Finland; 2 Department of Public Health, Babes-Bolyai University, Pandurilor 7, Cluj-Napoca, Romania; 3 National Health Service, 80 London Road, London, SE1 6LH UK; 4 Unit for Health Promotion Research, Institute of Public Health, University of Southern Denmark, Niels Bohrs Vej 9, 6700 Esbjerg, Denmark

**Keywords:** Physical activity, Health equality, Health equity, Policy, Policy making, REPOPA, Europe

## Abstract

**Background:**

There is strong research evidence on the importance of health equity and equality for wellbeing in societies. As chronic non-communicable diseases are widespread, the positive impact of physical activity (PA) on health has gained importance. However, PA at the population level is far from optimal. PA depends not only on individual factors, but also on policies for PA in sport, health, transport, education and other sectors, on social and cultural factors, and on the environment. Addressing health inequalities and inequities in PA promotion policies could benefit from policy development processes based on partnership and collaboration between various sectors, researchers, practitioners and policy makers (= cross-sectoral, evidence-informed policy making). The objective of this article is to describe how equity and equality was addressed in PA policies in four EU member states (Denmark, Finland, Romania and England), who were partners in the REPOPA project (www.repopa.eu, EC/FP7/Health Research/GA 281532).

**Methods:**

Content analysis of 14 PA policies and 61 interviews were undertaken between 2012 and 2013 with stakeholders involved in developing PA policies in partner countries.

**Results:**

Even though specific population subgroups were mentioned in the policy documents analysed, they were not necessarily defined as vulnerable populations nor was there a mention of additional emphasis to support such groups from being marginalised by the policy due to inequity or inequality. There were no clear objectives and activities in the analysed policies suggesting commitment of additional resources in favour of such groups. Addressing equity and equality were often not included in the core aims of the policies analysed; these aspects were mentioned in the background of the policy documents analysed, without being explicitly stated in the aims or activities of the policies. In order to tackle health inequities and inequalities and their consequences on the health status of different population subgroups, a more instrumental approach to health equality and equity in PA promotion policies is needed. Policies should include aims to address health inequalities and inequities as fundamental objectives and also consider opportunities to allocate resources to reduce them for identified groups in this regard: the socially excluded, the remote, and the poor.

**Conclusions:**

The inclusion of aspects related to health inequalities and inequities in PA policies needs monitoring, evaluation and transparent accountability if we are to see the best gains in health of socially disadvantaged group. To tackle health inequities and inequalities governance structures need to take into consideration proportionate universalism. Thus, to achieve change in the social determinants of health, policy makers should pay attention to PA and proportionally invest for universal access to PA services. PA promotion advocates should develop a deeper awareness of political and policy structures and require more equity and equality in PA policies from those who they seek to influence, within specific settings for policy making and developing the policy agenda.

## Background

Health inequalities have been defined as differences in health status or in the distribution of health determinants between different population groups. Some health inequalities relate to biological variations, free choices in lifestyles or to environment and conditions mainly outside the control of individuals. Therefore, some health inequalities are unavoidable, whereas others can be influenced or changed [1]. Inequities occur as a consequence of differences in opportunities, resulting, for example, in unequal access to health services, nutritious food, adequate housing and physical activity (PA) [2].

The importance of addressing health inequities and inequalities in health promotion policies with an emphasis on PA has been gaining more support in recent years [3], behavioral risk factors, such as low physical activity being recommended as to be part of health and social policies [4]. However, the explanation of these inequalities in health is complex. They cannot be reduced to a single group of risk factors, but undoubtedly inequalities in behavioral risk factors, such as a sedentary lifestyle, constitute a substantial part of the explanation. Health related behavior can be positively influenced only by addressing health-related behaviors and socioeconomic and environmental factors in PA policies. In various studies that present data from the European countries, it has been shown that smoking [5, 6], excessive alcohol consumption [7], lack of physical exercise [8, 9] and obesity [10] are all more common in lower socioeconomic groups. However, the extent of the impact of behavioral risk factors on health differs between countries. A recent study in the European countries showed that up to 9% of mortality due to a lack of physical exercise was attributable to health inequalities among men and up to 19% among women [11]. On the other hand, 12% of mortality among men and up to 42% among women was attributed to health inequalities [12]. Therefore, the contribution of these factors to inequalities in health also varies considerably, suggesting that priorities for public health policy should also differ between countries, for example, by focusing efforts and resources on selected population subgroups.

As a consequence of health inequalities, low levels of PA are one of many threats to health, and thus have a strong negative impact on the health system and health budget [13]. In Europe, physical inactivity has become a leading risk factor for ill health. It is estimated that physical inactivity causes 5.5% of coronary heart disease, 6.8% of type-2 diabetes, 9.3% of breast cancer, 9.8% of colon cancer and 8.8% of all-cause mortality [14]. Eliminating physical inactivity in Europe would lead to a gain of approximately 0.63 years in life expectancy [3]. Through adequate interventions focusing on most deprived or at risk target groups, PA levels can be increased to provide benefits like reducing inequalities in both PA levels and health across population subgroups [3].

Despite growing attention, socioeconomic health inequalities (e.g. impact of education and income levels) remain one of the greatest challenges to health policy in Europe [15–17]. Inequalities in mortality and morbidity between people with a higher and a lower socioeconomic position have been documented in all countries with available data [18]. Over recent decades, relative inequalities in mortality between those with a lower and a higher education, and between those with a lower and a higher occupational class have increased almost everywhere in Europe [7].

In the Physical Activity and Networking (PHAN) study [3] low physical activity was found to be related to individual choice, intrinsic motivation and friends’ interests in young people, whereas in adults low socio-economic status was generally related to low PA. In addition, social and environmental barriers were important among some ethnic groups. Further, the PHAN study concluded that personal, social and environmental factors influence on PA in low socio-economic groups. Therefore to tackle low PA, interventions addressing cultures of the communities and building partnerships and cross sector collaborations must have an impact on environment, making it more conducive to PA. In addition, it has been documented that PA promotion programs should be long enough to have an impact. Research indicates that adults and older people from disadvantaged backgrounds, as well as some minority ethnic groups, engage less in PA and are harder to reach for the promotion of PA than others [3]. Persons with disabilities are another particularly vulnerable group, with an elevated risk of health problems associated with physical inactivity. Research also indicates that vulnerable groups, including, but not limited to, unemployed adults or adults with low incomes, persons with disabilities and housewives, especially those with small children, are particularly hard to reach and should receive special attention [3].

The Commission on Social Determinants of Health has outlined determinants that interact and affect health equity and wellbeing, including structural drivers like policies, governance and societal norms and values [19]. The commission further developed three broad approaches to reducing health inequities:(1) targeted programs for disadvantaged populations; (2) closing health gaps between worse-off and better-off groups; and (3) addressing the social health gradient across the whole population. Policies to improve the availability, affordability and acceptability of PA for the most vulnerable groups can contribute to reducing their risks of disease, alongside policies in other areas. Based on these approaches, policies for improvement in equity in PA could also be developed. Such policies should focus on reducing ill-health suffered by different social groups [17].

In line with ensuring equity in the health sector [2], equity in PA policies should also be considered for addressing people’s needs, distribution of opportunities regarding PA services, choices and access between and within population groups, but also for providing fair and justifiable access to resources for health, such as PA. Features for equitable PA opportunities could be described as general access to PA services, access to counseling and an instructor, access to public and/or private PA services or places of PA. However, context varies and defines these opportunities and choices. In this study, equity in health means fairness of decision makers in guiding the distribution of opportunities for wellbeing, such as PA. The global strategy of Achieving Health for All [2] underlined that all people should have equal opportunities to develop and maintain their health, through fair and just access to resources for health, such as access to and possibility for PA [20, 21].

The aim of this study is to describe how equity and equality issues (e.g. selection of target population subgroups for PA policy; the policy description of values, priorities and political agenda; the justification of the need for specific PA policy) were addressed in selected PA policies from four European countries. The article discusses PA as an objective or means to create equity and equality in health. By documenting the use (or lack of use) of equality and equity arguments in selected PA policies in European countries, we strive to contribute to a better understanding of the ways in which policymakers consider and address (or do not consider) these issues in order to develop evidence-informed interventions to improve equality and equity in PA policies, as a mean to reduce health inequalities.

## Methods

### Design

This study was conducted as part of the ‘Research into Policy to enhance Physical Activity (REPOPA) project within the European Commission’s FP7 funding framework [22, 23]. The study aimed to gain an insight into the consideration of equality and equity issues and of vulnerable groups in selected PA policies. The study included PA policies from four REPOPA countries, Denmark, Finland, Romania and England. Content analysis of selected policy documents was conducted followed by stakeholders’ interviews to identify if equity and equality issues were considered in the selected policy documents.

The REPOPA project aimed to integrate scientific research knowledge, expert know-how and real world policy making process and to have academic institutions producing evidence as collaborating partners. Also, the project included organisations involved in the development and implementation of public health policies, as this was seen as an important component in the process of to bringing in their interests, values and priorities (Table 1). Further, to understand cultural and other contextual challenges, collaborating partners for this study came from four European countries representing different European regions [for further details see 22,23]. In each of these four countries, a national research team selected at least one local, regional and national PA related policy, if available, conducted documents analysis and stakeholder interviews following a commonly agreed protocol and reported the results.

**Table 1:** Country, title of analyzed policy paper, subpopulation specificity of the policy paper, timeline and number of interviewees

Title of policy paper and timeline	Primary responsible authorities of the policy paper	Overall vision of the policy	Objectives related to HEPA
Denmark 17 interviews			
Regional: The regional action plan – Region of Zealand 2012–2015	Region of Zealand	A broad-based functional regional policy and serves as an overall operating document for the development of social wealth, introduction of a green region theme, increased education level, tourism, and entrepreneurship, improved health and welfare services and infrastructure and transportation systems.	The policy touch areas where sport and exercise will play a role and be promoted, like linking HEPA to cultural activities with the health care system and ensure more recreational routes for cyclists.
Local: Copenhagen City’s Public Health Policy – Long live Copenhagen 2011–2014	City of Copenhagen	A public health policy targeting all citizens of the City of Copenhagen with special attention to vulnerable groups. The policy covers health in general, while physical activity is highly prioritized throughout the policy document.	The policy document included goals for physical activity, smoking, alcohol and self-assessed health. For physical activity the goals were to increase physical activity among young people and the general population.
Local: The Sports and Physical Activity Policy of Esbjerg Municipality2011-2014	Esbjerg Municipality, Department of Children and Culture	The vision was that through innovation and flexibility, the citizens would in every phase of their life be connected to sports and physical activity, which would provide energy and health to people.	The policy was divided into the following overall goals: physical environment, visibility, health promotion, non-elite sport, talent development and elite sport. This policy is an umbrella paper from which specific action plans are developed.
Local: The Health Policy of Odense Municipality 2012	The Municipality of Odense	The guiding vision was ‘to play is to live’. Also specific sector plans were created among these on sports and recreation. For the local health policy the overall ambition was to increase life expectancy free of disability or illness for everyone at all ages.	The health policy contained six focal areas: Healthy workplace and workforce; Mental health promotion; Reducing health inequalities; Strengthened efforts in relation to chronic diseases; Promoting health via the built-environment; Making healthy choice obvious and possible.
Finland 15 interviews			
National: Development of health enhancing physical activity and nutrition 2008–2011	Ministry of Health and Social Affairs; Ministry of Education; Ministry of Forest and Agriculture; National Sports Council, Advisory Board for Physical Activity	Increase the number of people for HEPA; increase number of people eating according the recommendations; decrease overweight and obesity and other nutrition and low HEPA related health problems; strengthen health-enhancing nutrition and HEPA practices in low socioeconomic groups.	Increase physically active lifestyle by increasing opportunities for physical activities in daily life; improving participation and a sense of community, enlarge equal opportunities for physically active hobbies; promote physical activities in day care, school and among students.
National: Promotion of physical activity 2009–2012	Ministries of Education, Social Affairs and Health, Environment and Finance; Association of Finnish Municipal and Regional Authorities, National Institute for Health and Welfare, Sport organizations and projects	Provide equal opportunities for physically active lifestyle and experience of communality through physical activity; improve understanding of physical activity as an essential part of wellbeing; safeguard conditions for physical activity; and make sustainable choices for physical activity.	Promote physical activity as a way of life by increasing opportunities for everyday physical activities at various stages in life. Improving preconditions for sport activities that promote participation and communality and by nurturing comprehensive equality in physical activity; development of favorable conditions, competence-building and measures to improve the prerequisites of non-governmental activities; reduce differences in physical activity and to support physical activity among the least advantaged population groups.
National: Strategy for Walking and Cycling 2020 2011–2020	Ministries of Transport, Communications and Environment, Finnish Transport Agency, centers for economic development, transport and the environment, cities and municipalities, the third sector	Make walking and cycling as normal and valued ways of getting around; walking and cycling more popular among all groups of people, both in cities and in rural areas; decrease health problems attributable to a lack of exercise from childhood to old age.	Promote walking and cycling to work and school and to access in public transport; provide a pleasant and safe physical environment; provide a variety of experiences and opportunities for social interaction; increase trips made on foot or bicycle by 20% and increase appreciation and motivation for walking and cycling of short distances and pleasant and safe surroundings.
Regional: Päijät-Häme Regional Health Enhancing Physical Activity Plan 2009–2020	Päijät-Häme Region, Municipalities in the region, sport and physical activity organizations, national welfare network	Provide conditions, services and know-how for health-enhancing physical activity.	Establish Regional Council for health-enhancing physical activity; develop for municipalities an action program on HEPA; Regional HEPA counseling as information sharing and motivation; development of HEPA conditions and services network; training and know-how development in HEPA.
Local: Lahti health enhancing physical activity strategy 2007	City of Lahti; technical and environment branch; social and health services branch; education and culture branch; physical activity.	Build permanent services and operation models in cooperation with the various fields of operation to promote prevention, individual’s own responsibility and health-enhancing physical activity among citizens.	Increase active lifestyles, communality and wellbeing; increase knowledge and skills on HEPA and its impacts and possibilities for health; train social and health sector employees to promote motivational interviews to enhance HEPA; invest in risk groups for HEPA and develop a cross-sector approach for HEPA with other partners.
Romania 11 interviews			
National: Movement for health 2003-ongoing	The Prime Minister of the Romanian Government; Ministry of Education and Research.	Contribute directly to improving Romanian population health status through physical education and sport.	Each sports facility that is administered by public authorities will be made available for recreational physical activities for a minimum of two hours, for at least three times per week for every citizen; periodical sports events for the general population; programs and actions for children and young people developed.
National: Sport for all 3^rd^ Millennium Romania – A Different Lifestyle 2001-ongoing	Romanian Federation Sport for All; Ministry of Health and Family actions; Ministry of Youth and Sport (currently Ministry of Education, Research Youth and Sport – National Authority for Sport and Youth)	Promote sport for all and health, education and recreation as a part of the lifestyle of the Romanian population. Some of the subprograms actions developed each year were: Fun-Sport, Old-Sport, Baby-Sport, Rural Sport, A chance for everybody.	Improved partnership between Government structures, economic agents and civil society in order to achieve the fundamental goals, such as guaranteeing the right of everyone to free access to practice recreational physical activities, form positive mentalities and attitudes at all population levels, ensure necessary conditions like human resources, logistics, management and quality services for practicing leisure time physical activity in organized settings or independently, in a safe and clean environment, and transition from centralized planning and develop local programs based on tradition, needs and preferences of the population.
County: The protocol for organizing sport activities for children in Cluj County – Cluj Champions 2011–2012 school year	Cluj County Council Cluj School Inspectorate Cluj Youth and Sport Department	Increase PA levels in Cluj County school aged children population	Contributing to the healthy physical and psychological development of the children participating in the competitions organized within the framework of the policy
England 12 interviews			
National: Places People Play – delivering a mass participation sporting legacy from the 2012 Olympic and Paralympics Games 2011–2015	Minister for Sport and the Olympics, and a multi-agency collaboration between Sport England in partnership with the British Olympic Association, the British Paralympic Association and with the support of the London Organizing Committee of the Olympic Games.	The initiative aims to deliver a mass participation sporting legacy from the 2012 Olympic and Paralympic Games	Investing in regionally significant multi-sport facilities; modernize and extend clubs and opening up of local facilities for community sport; protect and improve hundreds of playing fields across the country; recruit, train and deploy 40,000 Sport Makers as the next generation of sports volunteers; motivate over 100,000 adults to test themselves in multiple Olympic and Paralympic sports; provide opportunities for teenagers and young adults aged up to 25 years to receive six weeks of coaching in the sport of their choice and guiding them into regular participation; disability program designed to inspire people with a disability to take part in sport
Local: Destination Hereford 2011/12–2014/15	Planning Department, Herefordshire Council	The local transport plan is guided by the vision of ‘A sustainable and integrated transport system which recognises the distinctive characteristics of Herefordshire’s rural and urban areas and provides for the transport needs of residents, visitors and the business community’.	The local transport plan is driven forward by delivering accessibility, tackling congestion, building safer roads and improving air quality. This is achieved among others means by increasing sustainable travel and developing cycling and commuting strategies. The local transport plan links with several key local policies, one of which is ‘improving health’, with one of its aims being to contribute to encouraging more active lifestyles

### PA policies

Fourteen PA policies from four European countries (Denmark, Finland, Romania, and England) were included for analysis in this study. The policies focused mainly on PA, health, transport, nutrition and regional or local development, including health and physical activity concerns. The responsible authorities for these policies were ministries and regional and local authorities, in cooperation with various stakeholders.

The aim of the selected policies was to increase physical activity of the overall population (and/or of specific population groups) in order to enhance health. To tackle the inactivity, various specific behavioral and promotive actions were planned in various contexts and environments. Table 1 presents the titles of the policies, the main responsible authorities for each policy and the timeframe for implementation, as well as the overall vision and objectives of the policies.

### Documentation of the themes in PA policies and interviews

Through document analysis we identified the policy themes, such as the level of the policy, the focus of the policy, and target of the policy in relation to equity and equality towards various population sub-groups. Given the complex processes of policymaking, it was decided to carry out a content analysis of the policy documents followed by stakeholders’ interviews. This two-step approach was done to first identify how inequity and inequality issues were integrated into policymaking processes and then to discuss and confirm identified issues during interviews.

### Interviews

Purposeful sampling was used to identify and select interviewees. This involved identifying and selecting individuals who were especially knowledgeable about or experienced with PA policy making within the policies included in the analysis [23–25]. A total of 61 stakeholders were interviewed (Denmark 17; Romania 11; England 12; and Finland 15 stakeholders). Interviewees were policy makers, researchers, public sector officials and other influential stakeholders who were directly involved in the policy making process of the policies selected for analysis. Interviewees were contacted by the national research teams and provided with basic information on the project. Consent forms were administered in national languages of the partner countries. Semi-structured interviews were undertaken to further explore the equity and equality issues in PA policies, using a common interview guide across all participating countries [23]. The interviews were conducted by researchers from the respective countries in their native language. Each interview had a duration of 60 to 90 min and where additional consent was granted, the interviews were audio-recorded (notes were taken if this consent was not given). The interviews were then transcribed [23].

### Content analysis of policy documents and interviews

In the content analysis stage, the policy documents were reviewed by the REPOPA country teams to identify equity and equality issues. This also gave an opportunity to identify broader political forces (for example stakeholder positions) influencing policy decisions and to define how equity and equality issues were considered in the policy making process.

The content analysis of PA policy documents and the interviews followed the approach of Ritchie and Spencer [26], which is focused on case and theme based approach. The analytical tool uses a framework, which follows interpretation approach by ordering data and involving thematic analysis, typologies and explanatory analysis. The REPOPA team prepared a common content analysis guideline of policy documents to be used by all partners. The common guideline covered the policy-making phases; selection of policies; the process description of the policy analysis and instructions for the PA policy analysis; the focus of analysis in relation to goals and processes; a schematic example of the analysed text of a policy as well as equality and equity and population groups.

Thereafter, the findings were reported in English and pulled together into one report. The implementation phase of the policies was not included in the analysis, as it was out of the scope of the study.

Ethical clearance was obtained in accordance with the requirements of each country. The REPOPA Coordinator developed an Ethics Road Map and Ethics Guidance Document to coordinate varying national ethics clearance procedures in partner countries. Ethics clearance was done in each country according to country-specific regulations and procedures (for details see [27]). Irrespective of the country requirements, the informed consent of all participants was obtained. Ethical Committees involved per country: Ethics Committee of the Region of South Denmark and the National Data protection Agency; Research Ethical Committee of the National Health and Welfare Institute in Finland; Ethics Committee of the University of Babes-Bolyai in Romania; and the Ethics approval by the Research Councils in England. The ethical clearance papers were approved by the European Commission before the start of the project. The European Commission had oversight of the whole project ethics.

## Results

### Policy level

The analysis included six national policies (one from the England, three from Finland and two from Romania), three regional policies (one from Denmark, Finland and Romania) and five local policies (one from England and Finland and three from Denmark) (Table 2). The analyzed PA policies differed in their style of presenting health equity and equality on national, regional and local levels, as the purpose of each policy was not necessarily focused on inequities or inequalities. Nevertheless, the policies served similar functions in their contexts, most of them representing examples of symbolic policies that may eventually lead to political initiatives and implementation programs.

**Table 2:** Level of physical activity policies and the main topics of the policies

National policies	Regional policies	Local policies
England: Olympics		Denmark, Finland: Physical activity (Esbjerg, Lahti)
Finland: Nutrition, (health enhancing) physical activity, walking and cycling	Denmark, Finland: Health enhancing physical activity, prevention	
Romania: Physical activity and health, infrastructure	Romania: Young people and physical activity & schools	Denmark: Health policy (Copenhagen, Odense)
		England: Transport

### Policy focus

PA policies analyzed presented how governments wished to express their concerns and intentions related to PA promotion in the population. In policies focused on health promotion, the main concerns were the achievement of population health outcomes, which could be influenced by how the health system was organized and funded, but mostly by enabling people to expand their capabilities and develop self-reliance in health and health promoting behavior (Table 1). This encompassed places where people lived, such as local communities, homes, workplaces and playing fields, and people’s access to resources for health, and opportunities for empowerment.

### Policy target population

In the analyzed PA policy documents various subgroups were considered as policy targets. Such groups included children (preschool, school age children), young people (teenagers, students), professional groups (teachers), older people and other specific groups (psychiatric patients; people with disabilities; rural residents; immigrants; the unemployed; the poor) and the private sector as collaborating partner in the PA policy process.

Our study looked at the target groups of various policies, with different main focuses (e.g. health promotion, physical activity, transportation) and at different levels (e.g. national, regional, and local). The results showed that the target groups of the policies varied and that the countries and policies were not comparable. The study presented a picture of how policies can focus on certain groups of people or alternatively approach a whole population to promote PA. The transport policies and Olympics policy focused on the many ways to arrange PA possibilities and provide opportunities to citizens to be active and continue their activities during their life span, whereas the other policies analyzed were more focused on the public health sphere to encourage PA.

### Country specific results

#### Target populations of policies

In Denmark the analysis of PA policies and interviews identified the target groups to be the general population, students, children, young people, and older people. Various vulnerable groups with detailed sub-groups were mentioned in the city policies of Odense and Copenhagen, but no specific mentions of low socioeconomic groups were included in these policies.

The analysis of Finnish PA policies and interviews identified the target groups according to stages of life, such as people of working age, children and young people, and aged population. Also, the needs of mobility impaired people, unemployed, students, professionals and people in sparsely populated regions were included in these policies.

In Romania the analysis of the policies identified the general population, preschool children, students and teachers, the population in the rural areas (rural sport), women (women-sport), seniors (ageing population-sport), and Romanians worldwide (diaspora) as target groups for the national sport for all promotion policy.

In England, the policy for places where people play mentioned several target groups such as sports clubs, national governing bodies, sports volunteers, coaches, club leaders, young people aged 14–25, people with disabilities, males and females, people from black and minority ethnic groups, and anyone over the age of 16 years. The Herefordshire transport policy mentioned its target groups as Herefordshire’s residents, those wanting to change their existing travel habits away from a car to more active modes of travel, children, school and businesses, schoolchildren, adults and pedestrians and cyclists.

### Considerations of equity and equality in PA policies

When looking at equity and equality issues in the policies, we looked at how the terms were mentioned as preconditions for health, specifically for low socioeconomic groups and for people with low education level, as well as the aims to provide possibilities to achieve better health through PA.

In the Danish language, equality and equity are terms that are used in parallel. In the Danish policies analyzed equality was used as an overall term. In Denmark, in the policy for the region of Zeeland, equality was not discussed. However, due to the relatively large geographical size of the region (containing 17 of the 98 Danish municipalities) differences in the level of education, income, and health across the region were mentioned as an issue of concern. In the policy concerning the city of Copenhagen, variations in inequality in health across districts were mentioned. The focus on inequality in health, and also in PA, was a part of the “Long Live Copenhagen” policy document, which presented several arguments to support the importance of focusing on equality in health, though without any references to support the arguments. The policy emphasized the importance of initiatives targeting citizens living in districts with a high proportion of residents with unhealthy lifestyles, sickness and ill‐health. The interviews confirmed that with the support of health centers in low resource districts, initiatives were developed to target Copenhageners who do not reach out for help regarding their own situation. Examples of initiatives delivered at the health centers were consultations about exercise, strengthening of social networks, and the creation of opportunities for play and movement. The purpose of such initiatives was to create more equality in public health, by significantly improving the health of Copenhageners in districts with a high level of inequality in health. In most cases managers and politicians decided that inequality across geographical areas should be highlighted.

In Odense, Denmark, equality issues regarding PA were particularly recognized in the goal of the policy document concerning urban spaces. Urban planning was aimed at enhancing physical activity among all citizens by increasing accessibility, active transport, green areas and diversified activities in a safe and informal public space, as well as in deprived neighborhoods. On the long run, the municipality hoped that a healthy framework will decrease lifestyle-related diseases among citizens. Equality in access and opportunities for healthy living could narrow inequality in health. Creating a publicly available framework for a healthy lifestyle was stated in the goal; “the easy choice is the healthy choice”, and contained elements that promote equality for PA. This latter goal specifically mentioned children and youth as an essential group to focus on, because healthy habits are often adopted in childhood. No direct reference to research evidence was presented that justified the chosen goals and subgroups. One of the main goals “greater equality in health” in the health policy stated support for the equity issue. Inequity in health is one of the political goals, which was considered both strength and a challenge.

In Esbjerg, Denmark, the policy itself did not mention equality. However, according to one interview with a politician, it was stated that politicians agreed to uphold and distribute equality and fair conduct to all. Nevertheless, the interviewees confirmed that, during the policy process, there were discussions on equality. For example, in the Sport and Physical Activity Policy it was discussed whether to select a life cycle approach, and how to include a variety of stakeholders in the policy-making phase and in the implementation phase. The life cycle approach was not integrated into the final policy due to resistance from the local sports clubs, who were the primary stakeholder, since they were not able to translate this approach into their own organizational objectives.

In Finland, in the sport and PA policy document it was emphasized that sport policy should promote the wellbeing, health and functional capacity of the population at different stages of life with an emphasis on child and youth sport. The aim was to strengthen the prerequisites of local activity, inclusive equality and the position of sports as a basic local service. The sport and PA policy underlined that everyone should have equal opportunities to pursue a sportive way of life and to gain positive experiences and a sense of communality through sports and PA. The arguments for equity were joy of life, health and wellbeing, and functional capacity at different stages of life; emotions and shared experiences; having equal opportunities, experiences and a sense of communality. In addition, the policy stated that the public sector offered motivations and promoted equality in sports, while NGOs promoted the idea of social capital and active citizenship through their actions. The public sector measures were geared to influencing the underlying reasons for physical inactivity, to support citizens’ choices in PA and to promote equality. Society, communities and individuals should commit to a shared vision of the potential of sport as a factor for wellbeing, incentives targeted to certain population groups, and full consideration of regional, linguistic and gender equality. However, no specific research evidence or budgetary allocation for these choices was presented.

According to interviewees in Finland, equity was reflected in all the policy issues as the responsibility of the public sector to produce equal and achievable services for all. However, it was admitted that taking equity into consideration was different depending on the municipality, with different levels of formal concern and different activities among the actors regarding equity. Also, equity was understood to mean that when taking into account the whole population, one actually does not pay attention to any particular person or group. One interviewee stated that “It seems that sports have ‘higher-middle-class features’ as the general population does not have enough funds to take part in sports through the sports associations. Sports became a question of economic status.” According to the interviewees, lack of PA and sports have intensified the economic polarization between population groups.

In Finland, Health Enhancing Physical Activity (HEPA) and nutrition policy specifically underlined the reduction of health inequality differences between population groups. The main concern mentioned in interviews was that the schools and student health services were expected to ensure that youth are in an equal position irrespective of their municipality of residence and the education institution that they attend.

In the Finnish Walking and Cycling policy, equity was mentioned as economically advantageous choices for individual citizens. Walking and cycling were considered to promote people’s equal possibilities for mobility and equality, particularly among those who do not drive a car. Based on interviews, equity was discussed and considered to be a self-evident issue in the policy making process. However, the policy was aimed at making walking and cycling equal to other modes of transportation and at enabling everyone the right to walk and cycle in equal circumstances.

In Finland, the policy of the Päijät-Häme region referred to equality in constructing pedestrian and bicycle routes, commuting safely to school or work by foot or bicycle and providing people with opportunities to exercise in their leisure time. Furthermore, pedestrian and bicycle routes were considered to promote safe outdoor activities for older persons. Pedestrian and bicycle routes were considered as local sports venues, which were suitable for almost anyone and would increase equality and interaction between people. According to interviews, people in lower socioeconomic groups should get PA close to their everyday life to prevent isolation and loneliness.

The policy of the City of Lahti did not mention equity or equality issues specifically. Some differences (especially between men and women) in the amount of PA inside subgroups were presented. Interviewees stated that equity was internally structured in policy and that it was discussed as a principle that everyone should have a possibility to access and participate in PA services.

In Romania, in the Movement for Health national policy, equality and equity issues and research or other evidence to legitimate these problems were not presented at all in the policy document. Although preschool children and (pre-university) students were addressed separately by the policy, they were not defined as vulnerable groups. According to the interviews, generally, in the policy making process, other types of evidence such as population preferences, traditions, as well as resources available at local level were the most important contributing factors in choosing the target groups. No scientific data was used to support the need for targeting these population subgroups (in relation to other population subgroups). Also, given the fact that the main purpose of this national policy was to develop sport infrastructure (i.e. 400 sport halls throughout the country), it is not clear, from the policy documents or the interviews, what arguments underpinned the decision regarding where these sport halls were most needed-in terms of equity and equality or in terms of access.

In Romania, the national policy Sport for All-Romania 3^rd^ Millennium, a Different Lifestyle did not mention specific equity or equality issues in the policy description. However, in the methods sections of the policy document it is stipulated that the subprograms will be “elaborated for different population structures taking into consideration the following indicators: age, occupation, ethnicity, deprived groups [no definition and types of deprived groups are included], diaspora”. No mentions were made in regards to the evidence behind choosing these specific population subgroups as targets for the subprograms, nor were these subgroups presented as vulnerable populations. In the interview phase was found that the target populations for this national program were chosen after considering the research evidence gathered from “The fitness potential of the population” study. The study was conducted nationally by the Ministry of Education within the school-aged population (although no specific results from this study could be identified in the policy document or during the interviews). As well other types of evidence, such as population (expressed) needs, previously implemented programs, on-site observations were used in the policy making. Different age groups, disadvantaged population subgroups, such as those in rural areas, have been thus considered. One of the interviewees mentioned that “Information from the territory regarding needs and problems, as well as a technical analysis” have contributed to the choice of the target populations needed for each of the subprograms of this national program.

In Romania the county (regional) policy Cluj Champions-The protocol for organizing sport activities for children in Cluj County did not mention equality and equity issues in the policy document. Based on the interviews with stakeholders, the urban/rural and male/female criteria, and with or without physical or mental disabilities, were considered when developing the activities to be organized in the implementation phase of this partnership policy (i.e. sport competitions for school-aged children on different sport branches were organized as a result of this partnership between the County Council, the County School Inspectorate and the County Youth and Sport Department). Hence, the planned competitions included boys and girls from urban and rural areas from Cluj County, with and without physical and mental disabilities. Of course, there were separate competitions organized for each of the aforementioned population categories, with the exception of urban/rural, which participated in the same competition for each sport branch (e.g. football, athletics).

In England, the Places People Play program did not present equity issues, except in regard to equity between male and female in some target groups. In England, the Herefordshire transport policy did not make reference to equity or equality issues, but mentioned improving conditions for vulnerable road users e.g. people with disabilities and children. The needs of the dispersed rural population and increasingly older population were also mentioned.

### Common aspects

None of the policies included into the analysis explicitly mentioned the issues of reaching equality and equity as on the policy focus. Most of PA policies analyzed in this study focused on the whole population and targeted one or more subgroups in more specific policies. To reach low socio-economic groups and provide equity in access to PA services requires more focus on longer term programs in PA to have an impact. Many of the policies are in force and implementation has been pending on elections and political powers in the government. However PA promotion is not pending on political changes, but lifetime encouragement and creating environment and culture for active lifestyle to bring the monetary and human resource savings for the societies.

None of the policies specifically addressed equity and equality in PA in relation to population groups, services or interventions. However most of the policies had some components of equity and equality in the policy documents, but they were not specifically underlined as such. Even some of the policies that did not mention equity and equality were reported to be discussed in the policy development process or in the programs for implementation. Though equity and equality were not directly addressed, they were considered to different extents in different contexts in Denmark, Finland, England and Romania.

## Discussion

While the importance of PA for health is well established, equal and equitable provision of PA services have not yet been the focus of analysis of PA policy studies. The few studies on equity and PA have focused on PA levels among population groups ([28], for example), but not on the root causes of limited access or on accessibility to PA possibilities and services in various population groups (i.e. disadvantaged ones). The equity and equality in PA needs advocacy planning and action to make the issue to be considered in policy making and addressed in PA policies.

The study of PA policies in the four European states showed that the consideration of equity and equality in PA policies varied in depth and in relation to various population groups and vulnerable groups. However, except some policies, that focused on only one population group, most PA policies analysed in this study focused on the whole population and targeted one or more subgroups, which yields similar results as in Christiansen et al. [29]. The inclusion of some population groups as policy target was not evidence informed. Therefore it’s not known if those population groups were the most in need for more allocated resources to increase PA levels. In addition, it was not clear if the financial allocation and the inclusion of some population groups as target in a policy covered the actual PA needs of those population groups. Therefore the policy in relation to vulnerable groups and promotion of equity and equality were more symbolic than instrumental.

A well-defined and effective PA policy in relation to health equality and equity issues has never been more important or relevant than now, due to the sedentary lifestyles and health inequalities and inequity present in many states [3]. Further, only a few policy documents on promoting PA have acknowledged the need to consider socially disadvantaged groups as a priority and have indicated a need to integrate this into mainstream policies [3]. Various health problems are potentially avoidable and governments have tools to influence population health and change individual behaviours in PA, directed both ‘upstream’ at some of the underlying causes of low PA and poor health, as well as ‘downstream’, at physical inactivity and poor health behaviours as they appear. According to the PHAN study PA interventions should be built into the culture of the target communities, be culturally sensitive and develop cross sector cooperation between sectors consistently. However, to achieve the desired impact, work with disadvantaged groups has to be local and focused to build PA as a norm in the communities [3].

The combination of taxation, legislation and health information remain the core components of any strategy to influence health promotion and especially PA [30]. To foster and strengthen PA in various policies it is important to assess the identity of political actors, such as leaders, interest groups, and various professionals.

In seeking to increase inclusion of PA into various policies, among the important elements are an understanding of the ideologies, available resources, and potential opportunities to influence the policy agenda during policy making [31–33]. The role of political actors within the complex interactions of various political, economic and social institutions and policies, and the effect of dominant political ideas also help explain the success, failure or promise of policy options for PA [34, 35].

Recently Carey et al. [36] presented a framework for the application of proportionate universalism. Distinctive policy objectives, such as promoting PA across population group needs, such as for children, people with disabilities or women, can be applied as an opposition of universalism in relation to the objectives of PA for the general population. Based on Carey et al. [36] positive selectionism can be used in relation to children, youth and specific professionals at their workplaces. The working-age population, students, families, older people and women could be considered as belonging to specific universalism, as these groups of populations constitute large parts of general populations. Within particularism, with distinctive social needs for PA, policies could focus on people with physical and mental disabilities, victims of violence or immigrants or on people in lower socioeconomic groups.

Interventions that address the social determinants of health, like PA, have the greatest potential for bringing public health benefits. Action on these issues needs the support of government and civil society if it is to be successful [37]. The biggest obstacle to making fundamental societal changes is often not shortage of funds, but lack of political will; the health sector is well positioned to build support and develop the partnerships required for change in PA [38]. Bull et al. [39] argued that the presence of other relevant goals from different sectors highlighted the opportunity for joint action. However very few countries have a national multisector coordinating committee and many countries have challenges with partnerships on different levels of policy implementation.

Policies describing health inequities and inequalities and their causes are not always argued clearly in the policy documents, particularly in terms of how solutions and actions work differentially across the social gradient. Therefore, the integration of research evidence and collaboration with other sectors is essential. In particular, decisions regarding what services need to be tailored to which individuals needs to be made by the level of governance closest to vulnerable groups. In addition, local government and nongovernment organizations, embedded in local communities, are more likely than national governments to understand the needs of specific individuals and groups and how best to address them [40]. Thus, their engagement should be encouraged and supported through formal mechanisms and structures.

## Conclusions

This research provides evidence on the current policy context for selected areas of Europe. In general there is scant research analysing PA policies across nations.

Equity and equality as values were often in the background of policies for PA, but were not necessary explicitly addressed and elaborated on the policies-they were used rather symbolic than instrumental. However, policy makers in responsible organizations decided which population groups or on which aspects of inequalities the policies should focus on. Nevertheless, there is a demand for evidence-informed policymaking including PA policies. Often the socioeconomic circumstances were not mentioned as justification for the selection of population groups. On the other hand, some policies made a link between insufficient PA, the influence of social environment, context or the importance of focusing on particular population groups, such as children, young people and socioeconomically vulnerable people.

Both researchers and policy makers across sectors and policy levels need to consider developing good policy making practices, such as basing policies on evidence-informed approaches across sectors, which target equity and equality issues. Furthermore, PA policy in relation to inequalities and inequities need monitoring, evaluation and transparent accountability if we are to make the best gains for health and thereby decrease health inequalities and inequities.

Proportionate universalism suggests that health interventions should be universal, not targeted, but with a scale and intensity that corresponds with the level of disadvantage. However, in the implementation of any policy that is based on a degree of targeting in how resources are allocated, decisions should be made on who will be included and who will be excluded [41].

Low PA is one of the contributors of health inequalities. Therefore, tackling health inequities and inequalities requires governance structures to take into consideration proportionate universalism. Thus, to achieve change in the social determinants of health, policy makers should pay attention to PA and invest proportionally in accordance with the needs informed by (research) evidence for fostering universal access to PA services.

PA advocates should develop a deeper awareness of political and policy structures and discuss equity and equality with those they seek to influence, within specific settings for policy making and developing the policy agenda.

## Abbreviations

PA: Physical activity
REPOPA: Research into Policy to enhance Physical Activity
